# Treatment of pregnant rats with oleoyl-estrone slows down pup fat deposition after weaning

**DOI:** 10.1186/1477-7827-6-23

**Published:** 2008-06-20

**Authors:** Beatriz García-Peláez, Ruth Vilà, Xavier Remesar

**Affiliations:** 1 Departament de Nutrició i Bromatologia, Facultat de Biologia, Universitat de Barcelona, Av. Diagonal 645, 08028 Barcelona, Spain; 2 CIBER (Fisiopatología de la Obesidad), Instituto de Salud Carlos III, Spain

## Abstract

**Background:**

In rats, oral oleoyl-estrone (OE) decreases food intake and body lipid content. The aim of this study was to determine whether OE treatment affects the energy metabolism of pregnant rats and eventually, of their pups; i.e. changes in normal growth patterns and the onset of obesity after weaning.

**Methods:**

Pregnant Wistar rats were treated with daily intragastric gavages of OE in 0.2 ml sunflower oil from days 11 to 21 of pregnancy (i.e. 10 nmol oleoyl-estrone/g/day). Control animals received only the vehicle. Plasma and hormone metabolites were determined together with variations in cellularity of adipose tissue.

**Results:**

Treatment decreased food intake and lowered weight gain during late pregnancy, mainly because of reduced adipose tissue accumulation in different sites. OE-treated pregnant rats' metabolic pattern after delivery was similar to that of controls. Neonates from OE-treated rats weighed the same as those from controls. They also maintained the same growth rate up to weaning, but pups from OE-treated rats slowed their growth rate afterwards, despite only limited differences in metabolite concentrations.

**Conclusion:**

The OE influences on pup growth can be partially buffered by maternal lipid mobilization during the second half of pregnancy. This maternal metabolic "imprinting" may condition the eventual accumulation of adipose tissue after weaning, and its effects can affect the regulation of body weight up to adulthood.

## Background

The administration of oleoyl-estrone (OE) (a naturally occurring ester of estrone and fatty acids stored in adipose tissue) to rats at pharmacological doses, induces a transitory decrease in food intake, resulting in a noticeable decrease in body weight that is not paralleled by any concurrent losses in body protein [1]. Since rats follow this same pattern under different nutritional and physiological conditions [2,3] hints at OE as a ponderostat (set-point of body weight) regulator [4]. Although receptors other than the estrogen receptor mediate OE effects [5] its mechanism of action remains unknown, and involves pathways different from those activated by food restriction alone [6].

It is well known that the availability of food during pregnancy affects the postnatal development of their litter [7]. The consequences of starvation or food deprivation are well documented, in particular the high correlation between maternal energy restriction and adult obesity [8]. Obesity is related to insulin resistance, hypertension and dislipemia, factors that alone or combined decrease the life span [9]. These effects are mediated, in part, by oxidative processes linked to inflammation [10]. On the other hand, there are evidences that energy restriction is associated with longer life span in rodents [11]. Thus, it can be argued that the control of factors eliciting the onset of obesity can also help decrease morbidity and mortality. We have intended to determine whether OE-treatment may affect pregnant rat descendants' growth pattern, since OE reduces voluntary energy intake.

## Methods

### Animals

Female virgin Wistar rats from Harlan-Interfauna (Sant Feliu de Codines, Spain) were mated with adult males until impregnation (confirmed by the presence of spermatozoa in daily vaginal smears); the day of impregnation was designed as day 0 of pregnancy. The animals were maintained in the Animal Service of the University of Barcelona, under standard conditions (light 12 hours on/off; 22°C, and 65% humidity) and had free access to standard chow pellets (14.5% of protein, 4% of lipids, 4.5% of fiber, 53.9% of starch and 10% of free sugars)(Panlab, Barcelona, Spain) and tap water. All procedures were in accordance with those guidelines governing the use of experimental animals established by the European Union, and were approved by the Animal Handling Ethics Committee of the University of Barcelona.

### Treatment

On day 11 of pregnancy, a group of 6 animals was sacrificed and used as a reference (P11 group). The remaining animals were randomly divided into two groups (n = 12), one receiving a daily gavage of 0.2 ml sunflower oil containing 10 nmol oleoyl-estrone/g of body weight (OED, Barcelona, Spain) (OE-treated group); and the other receiving only the vehicle (Control group). The rats were kept in individual cages, and their daily food consumption and body weight were recorded. The gavages were administered from days 11 to 21 of pregnancy. On day 21, 6 animals from each treatment group were sacrificed (P21 groups). The rest were sacrificed on day 20 after delivery (L20 groups). 21-day fetuses (F21) and half of the 20-day old pups (PP20) were beheaded and blood and adipose tissue samples were obtained. The remaining PP20 pups were placed in collective cages and sacrificed on day 30 (PP30) after birth. The weight of pups was recorded daily after weaning, on day 20. Pregnant rats on days 11 and 21 were used in body composition analysis and energy balance calculations.

### Samples

All pregnant rats and their pups were killed by swift decapitation. Blood was recovered and allowed to clot; serum was frozen and stored at -80°C until processed. Samples of white adipose tissue (WAT) from different locations (perigonadal, retroperitoneal and mesenteric), and the interscapular brown adipose tissue (IBAT) were immediately excised, frozen in liquid nitrogen, weighed and stored at -80°C. Periovarian and epididymal samples from pups were combined and used as a single perigonadal sample. DNA content was measured using a standard fluorimetric method with 3, 5-diaminobenzoic acid (Sigma, MO, USA) using bovine thymus DNA as standard [4]. Since all mammalian cell nuclei contain the same amount of DNA, we were able to estimate the approximate number of cells in a given WAT (or IBAT) sample by dividing its DNA content by the mean DNA content of a cell (6 pg/cell) [4]. The mean mass of the cells in a given WAT site was determined by dividing the weight of the tissue by the number of cells it contained [4,12].

### Analytical procedures

Serum samples were used to measure glucose (Glucose HK CP Kit, Horiva ABX, Madrid, Spain), urea (kit B8035 from Menarini, Firenze Italy), triacylglycerols (kit 11528; Biosystems, Barcelona, Spain), total cholesterol (kit B7576; Menarini), protein [13], HDL-cholesterol (precipitating kit CH204 from Randox, Crumlin UK; and kit B7576 from Menarini), non-esterified fatty acids (NEFA) (kit NEFA-C; Wako, Richmond, VA USA), 3-hydroxybutyrate (kit 0907979; Boehringer-Mannheim, Mannheim, Germany), insulin (rat insulin RIA kit; Linco, St Louis, MO USA), adiponectin (mouse adiponectin RIA, Linco) and leptin (rat leptin RIA, Linco). All commercial assays were performed according to manufacturer's instructions.

### Balances

After sampling, the stomach and intestinal contents were discarded; the remaining rat carcasses were autoclaved, homogenized and used to estimate water, protein, lipid and energy content as previously described [2]. Briefly, water was estimated by differential weighing before and after desiccation at 110°C; protein was estimated from the N content (Kjeldahl, using the 1007 Digestor and 1002 Distilling Unit, both part of a Tecator Kjeltec System, Höganäs, Sweden) and conversion of the N content into a protein equivalent [14]; lipid content was estimated by trichloromethane: methanol extraction [15]; and total energy by using a bomb calorimeter (C-7000 Ika, Heitersheim, Germany). The final body composition was established from the percentages of body components measured experimentally and the estimated *in vivo *net body weight. For comparative balance calculations, we used the mean body weight composition values of the group killed at the beginning of the study (day 11).

The metabolizable energy content of the pellet food was estimated from the standard caloric equivalence of its assimilable components (154 g/kg crude protein, 605 g/kg carbohydrate, 29 g/kg lipid) and the assumed efficiency of the digestive process, giving a yield of 13.9 kJ/g. The total energy content of the pellet was estimated using the bomb calorimeter (16.5 kJ/g), which means that only about 84 % of the total energy contained in the pellet (including that from fiber) was taken up and used by the rat [16].

Statistical comparison between groups was carried out using two-way ANOVA and a post-hoc Bonferroni tests using the program Prism 4 (GraphPad Software Inc, San Diego, CA, USA). The unpaired Student's *t *test was used for specific comparisons.

## Results

**Figure 1:**
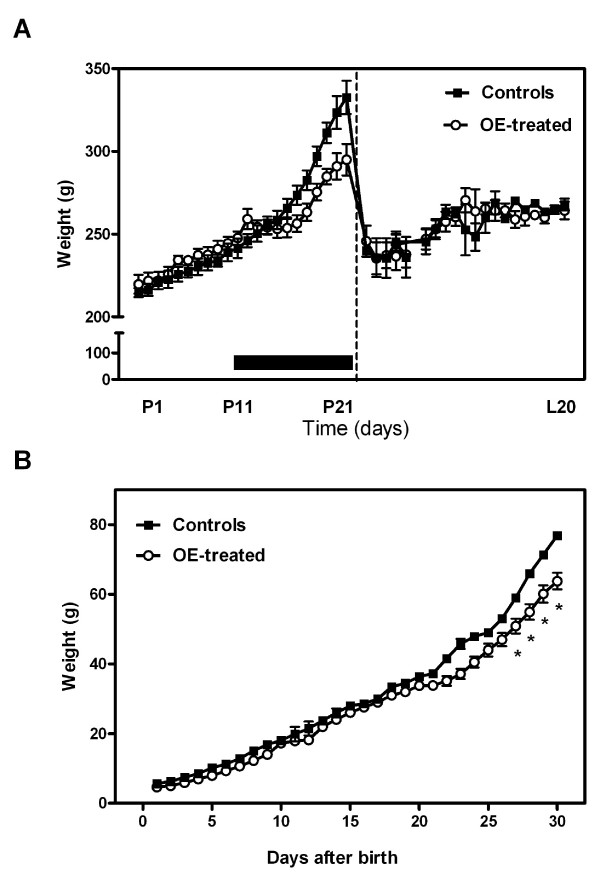
**Weight changes of OE-treated rats and their pups during pregnancy**. A) Weight changes in dams. The black bar indicates the period of treatment. P1, P11 and P21 refer to days 1, 11 and 21 of pregnancy and L20 refers to day 20 after delivery. Statistical differences: two-way ANOVA; factor time (P < 0.0001), factor treatment (P < 0.0001) and significant interaction (P = 0.0001). Bonferroni post-hoc test: the weights of treated rats showed no differences. B) Weight changes in pups from control or OE-treated mothers during pregnancy. Statistical differences: two-way ANOVA; factor time (P < 0.0001), factor treatment (P < 0.0001), significant interaction between factors (P = 0.0002). Bonferroni post-hoc test: * = P < 0.05. There were significant differences between groups from day 27 onwards.

In Fig. 1A are depicted the variations in weight of pregnant rats along time. Specifically, control rats increased their weight 118 ± 4 g from day 0 to day 21, whereas OE-treated rats increased 65 ± 4 g. After parturition, both groups' dams had the same weight. Fig. 1B shows that the pups from OE-treated rats had a growth pattern similar to that of controls from birth up to day 20. After weaning, the treated group slowed its growth rate, significantly diverging from controls from day 27 onwards.

**Figure 2:**
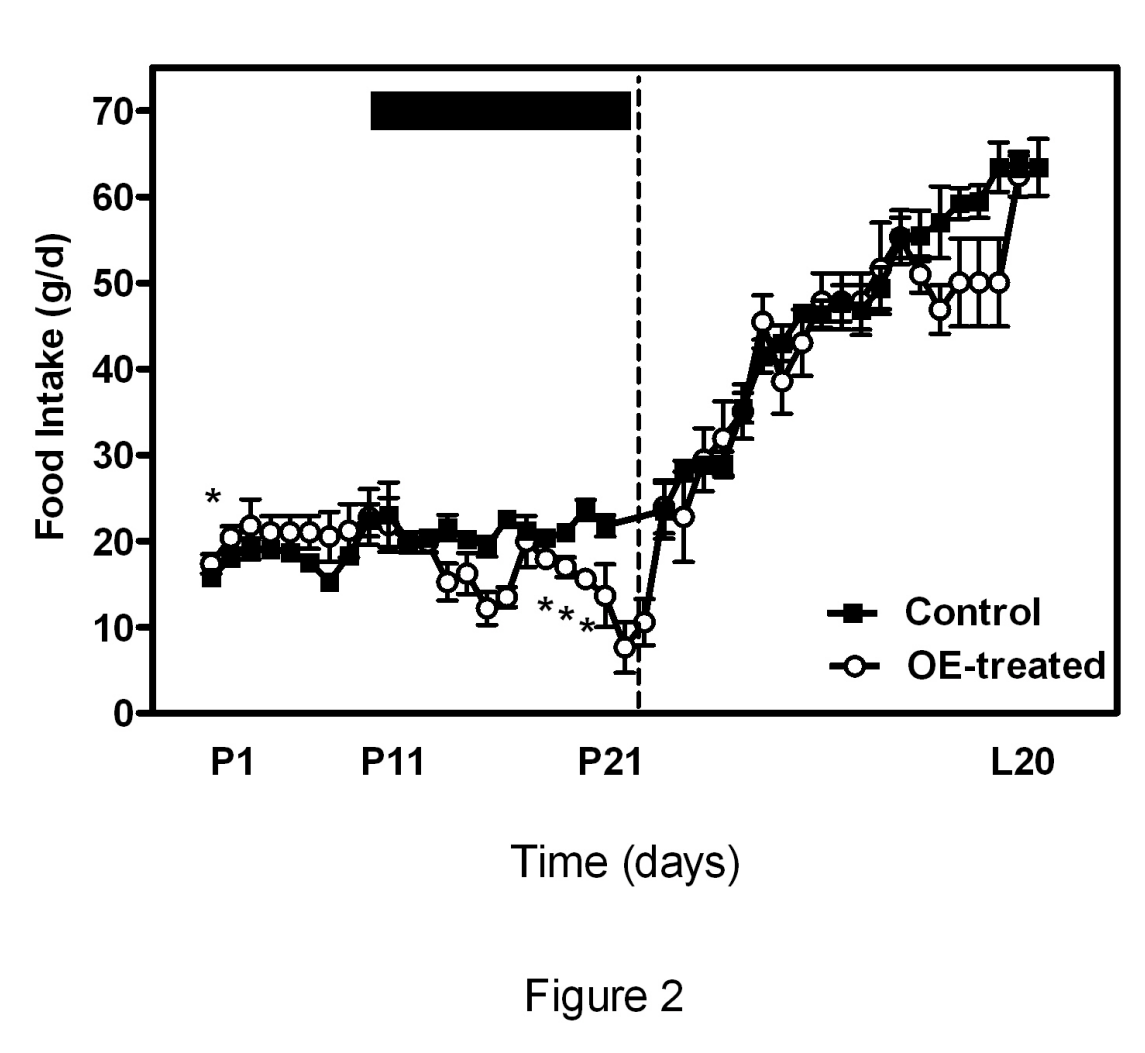
**Changes in food intake of rats treated with OE during the second half of pregnancy**. The black bar indicates the period of treatment. P1, P11 and P21 refer to days 1, 11 and 21 of pregnancy and L20 refers to day 20 after delivery. Statistical differences: two-way ANOVA; factor time (P < 0.0001), factor treatment (P < 0.0001), significant interaction between factors (P = 0.0003). Bonferroni post-hoc test: There were significant differences between the groups from days 1, 16, 17 and 19 after delivery (* = P < 0.05).

The changes in daily food intake caused by treatment can be seen in Fig. 2. Control animals ingested 1328 ± 62 g of standard chow pellets during the entire study period, whereas treated dams ingested 1193 ± 113 g (i.e. 89.8% of controls). There were significant differences between groups on days 1, 16, 17 and 19 of pregnancy.

Changes in serum metabolites during pregnancy and lactation period, both in dams and their pups are shown in Table 1. Transition from pregnancy to lactation induced significant changes in almost all serum metabolites in the dams, with increases in urea and cholesterol and decreases in protein, non-esterified fatty acids and triacylglycerols. OE treatment induced significant decreases in glucose and HDL-cholesterol in late pregnancy, and in ketone bodies and non-esterified fatty acids at weaning, whereas protein levels were increased on day 20 after delivery. Transition from fetal life to suckling pups brought significant changes (essentially increases) in all parameters tested, except for urea and 3-hydroxybutyrate. Pups from OE-treated mothers showed significant increases in protein and HDL-cholesterol and decreases in glucose on day 30 (PP30); the descendants from OE-treated dams showed lowered glucose values in fetal life and in ketone bodies on day 20, and increased protein values at weaning.

**Table 1:** Serum metabolite values in pregnant and lactating rats and their fetuses/pups.

		**Pregnant/Lactating rats**			
		P11	P21	L20	ANOVA
Glucose mM	C	6.81 ± 0.20	6.98 ± 0.52	7.77 ± 0.11	
	OE		5.58 ± 0.48 *	6.87 ± 0.07	
Protein g/l	C	64.4 ± 1.42	64.5 ± 2.13	53.2 ± 1.01	T, TxTR
	OE		63.4 ± 1.43	60.8 ± 2.16 *	
Urea mM	C	6.05 ± 0.32	5.29 ± 0.33	10.3 ± 0.41	T
	OE		5.61 ± 0.71	11.1 ± 0.98	
Non-esterified	C	0.21 ± 0.01	0.95 ± 0.04	0.62 ± 0.10	T, TxTR
fatty acids mM	OE		1.07 ± 0.04	0.41 ± 0.05 *	
3OH butyrate μM	C	93.2 ± 8.22	106 ± 15.3	90.1 ± 18.9	T
	OE		99.8 ± 9.42	38.7 ± 3.53 *	
Cholesterol mM	C	0.88 ± 0.06	0.83 ± 0.07	3.74 ± 0.23	T, TxTR
	OE		0.49 ± 0.06	4.11 ± 0.18	
HDL Cholesterol mM	C	0.44 ± 0.03	0.48 ± 0.08	0.34 ± 0.05	
	OE		0.30 ± 0.01 *	0.39 ± 0.06	
Triacylglycerols mM	C	1.25 ± 0.09	1.90 ± 0.04	0.52 ± 0.04	T
	OE		1.93 ± 0.08	0.57 ± 0.06	

		**Fetus/Pups**			

		F21	PP20	PP30	ANOVA

Glucose mM	C	4.01 ± 0.21	7.83 ± 0.11	8.84 ± 0.12	T, TR, TxTR
	OE	3.16 ± 0.12 *	7.84 ± 0.09	8.12 ± 0.12 *	
Protein g/l	C	24.7 ± 1.40	40.8 ± 0.94	44.2 ± 1.74	T, TR, TxTR
	OE	21.1 ± 0.40	49.6 ± 1.40 *	55.1 ± 1.21 *	
Urea mM	C	5.37 ± 0.83	5.79 ± 0.90	5.24 ± 0.38	
	OE	5.87 ± 0.70	4.36 ± 0.42	4.95 ± 0.58	
Non-esterified	C	0.24 ± 0.01	0.49 ± 0.06	0.59 ± 0.04	T
fatty acids mM	OE	0.21 ± 0.02	0.57 ± 0.11	0.64 ± 0.01	
3OH butyrate μM	C	288 ± 3.64	250 ± 7.31	91.5 ± 14.1	T, TxTR
	OE	297 ± 3.25	211 ± 15.9 *	108 ± 7.47	
Cholesterol mM	C	1.15 ± 0.06	3.46 ± 0.11	2.13 ± 0.09	T
	OE	0.98 ± 0.13	3.27 ± 0.18	2.07 ± 0.01	
HDL cholesterol mM	C	0.12 ± 0.02	0.15 ± 0.01	0.18 ± 0.02	T, TxTR
	OE	0.08 ± 0.03	0.15 ± 0.02	0.35 ± 0.08 *	
Triacylglycerols mM	C	0.27 ± 0.04	2.03 ± 0.01	1.67 ± 0.16	T
		0.41 ± 0.11	1.91 ± 0.07	1.52 ± 0.13	

C: control group; OE: treated group. Values are expressed as the mean ± s.e.m. of 6 animals per group. Statistical analysis of the differences: Two-way ANOVA (time (T), treatment (TR) and interaction (TxTR)) was performed.

Post-hoc Bonferroni test (comparison between C and OE): * = P < 0.01

In Table 2, the levels of serum hormones are depicted. A significant decrease in insulin and adiponectin progressed from late pregnancy to lactation. OE treatment decreased leptin and adiponectin during late pregnancy. Developing pups showed increased adiponectin both in control and OE-treated groups. OE treatment caused a significant increase in insulin at weaning and decreased leptin and adiponectin on day 30 (PP30).

**Table 2:** Serum hormone values in pregnant and lactating rats and in their pups.

		**Pregnant/Lactating rats**			
		P11	P21	L20	ANOVA
Insulin nM	C	0.38 ± 0.06	0.21 ± 0.04	0.18 ± 0.04	
	OE		0.18 ± 0.04	0.19 ± 0.04	
Leptin nM	C	0.17 ± 0.01	0.31 ± 0.07	0.09 ± 0.01	T, TR, TxTR
	OE		0.10 ± 0.01*	0.10 ± 0.01	
Adiponectin nM	C	348 ± 39.8	279 ± 12.3	96.8 ± 25.2	T, TR, TxTR
	OE		144 ± 7.97 *	95.3 ± 17.1	

			**Pups**		

			PP20	PP30	ANOVA

Insulin nM	C		0.06 ± 0.01	0.10 ± 0.01	T, TxTR
	OE		0.19 ± 0.03*	0.11 ± 0.01	
Leptin nM	C		0.10 ± 0.01	0.18 ± 0.02	TR, TxTR
	OE		0.12 ± 0.01	0.07 ± 0.01*	
Adiponectin nM	C		60.7 ± 6.98	195 ± 12.9	T, TxTR
	OE		69.3 ± 9.03	160 ± 9.93*	

C: control group; OE: treated group. Values are expressed as the mean ± s.e.m. of 6 animals per group. Statistical analysis of the differences: Two-way ANOVA (time (T), treatment (TR) and interaction (TxTR)) was performed.

Post-hoc Bonferroni test (comparison between C and OE): * = P < 0.01

Body composition of pregnant rats on days 11 and 21 of pregnancy, as well as the changes caused by OE treatment are depicted in Table 3. These changes were determined by comparing an ideal composition of each rat on day 11 (using the mean P11 body composition but their actual day 11 weight) with their measured composition and weight on the final day of treatment (P21). Our data show that OE treatment induced a limited increase in body weight, essentially a consequence of low lipid accrual. Since protein content did not change, and lipid content was reduced in OE-treated rats, the decrease in total energy content in OE-treated rats was a consequence of the decreased lipid content.

**Table 3:** Body composition of rats on days 11 and 21 of pregnancy.

		**P11**	**P21**	
			**Controls**	**OE-treated**
Body weight (g)		250 ± 5.8	335 ± 7.09	298 ± 10.6 *
**Body composition**				
Lipid content (g)		33.9 ± 0.67	45.4 ± 0.99	30.6 ± 1.32 *
Protein content (g)		43.3 ± 0.86	48.6 ± 0.97	47.9 ± 1.47
Energy content (MJ)		2.18 ± 0.04	2.63 ± 0.006	2.06 ± 0.09 *
**Changes in body composition**				
Body weight	Absolute (g)		85.2 ± 7.04	46.5 ± 4.91 *
	*Relative (%)*		*34.1 ± 2.82*	17.8 ± 1.61 *
Protein	Absolute (g)		5.24 ± 1.05	3.85 ± 0.71
	*Relative (%)*		*12.1 ± 2.38*	*8.89 ± 1.48*
Lipid	Absolute (g)		11.8 ± 0.67	-3.10 ± 0.37 *
	*Relative (%)*		*34.8 ± 1.15*	-9.14 ± 1.22 *
Energy (MJ)	Absolute (g)		0.47 ± 0.03	-0.17 ± 0.03 *
	*Relative (%)*		*21.5 ± 1.36*	-7.96 ± 1.30 *

Changes in body composition are shown, either in absolute or in relative values. The values are expressed as the mean + s.e.m. of 6 animals.

Statistical differences: * = P < 0.05 (OE-treated vs. controls).

Changes induced in the weight, cell number, and cell mass of different adipose tissue depots, both in the dams and their pups can be seen in Table 4. Although adipose tissue weights increased throughout pregnancy, during the transition to lactation they markedly decreased except for IBAT. The loss of WAT was parallel to decreases in cell number counts in all the locations, but also by increases in brown adipose tissue cell number. OE treatment reduced cell number in all WAT locations in late pregnancy, but not during lactation. In the pups, we observed the expected increase in different adipose tissue locations together with higher cell numbers in retroperitoneal and perigonadal sites, but only increased mean cell mass in the perigonadal site. The pups from OE-treated mothers showed decreases in the weight (and a tendency towards increased cell number) of mesenteric and perigonadal pads (day 30), and decreased cell numbers in retroperitoneal location.

**Table 4:** Adipose tissue weight, relative weight (% of body weight, bw), cell number and cell mass in pregnant rats and their pups.

		**Pregnant/Lactating rats**			
		P11	P21	L20	ANOVA
**Mesenteric WAT**					
Weight g	C	4.30 ± 0.33	6.31 ± 0.40	0.73 ± 0.11	T, TR, TxTR
	OE		3.43 ± 0.51 *	1.09 ± 0.17	
Cell number n × 10^6^	C	1076 ± 262	3382 ± 1415	408 ± 164	TxTR
	OE		483 ± 104 *	681 ± 289	
Cell mass ng	C	3.90 ± 0.86	2.28 ± 1.22	2.27 ± 1.23	TxTR
	OE		7.55 ± 0.46 *	2.88 ± 1.43	
**Retroperitoneal WAT**					
Weight g	C	3.47 ± 0.42	4.69 ± 0.79	1.19 ± 0.05	T, TR, TxTR
	OE		2.05 ± 0.20 *	1.36 ± 0.14	
Cell number n × 10^6^	C	228 ± 23.0	212 ± 34.5	87.4 ± 12.2	T, TR, TxTR
	OE		99.4 ± 7.13 *	89.9 ± 3.09	
Cell mass ng	C	14.5 ± 0.31	20.2 ± 0.81	14.1 ± 1.98	
	OE		21.2 ± 2.72	15.4 ± 1.32	
**Perigonadal WAT**					
Weight g	C	3.62 ± 0.42	7.44 ± 1.61	2.14 ± 0.61	T, TR, TxTR
	OE		3.58 ± 0.71 *	2.19 ± 0.45	
Cell number n × 10^6^	C	324 ± 30.3	432 ± 23.9	207 ± 27.7	T, TR, TxTR
	OE		243 ± 26.4 *	164 ± 28.8	
Cell mass ng	C	11.2 ± 0.78	18.7 ± 3.83	9.91 ± 1.74	T
	OE		14.1 ± 1.44	14.6 ± 3.65	
**Interscapular IBAT**					
Weight mg	C	330 ± 21.2	390 ± 91.2	340 ± 31.4	
	OE		391 ± 25.1	287 ± 26.6	
Cell number n × 10^6^	C	154 ± 13.9	92.9 ± 15.9	343 ± 4.21	T, TR, TxTR
	OE		115 ± 10.3	191 ± 66.8 *	
Cell mass ng	C	2.20 ± 0.16	4.21 ± 0.39	0.98 ± 0.09	T, TxTR
	OE		3.49 ± 0.28	2.31 ± 0.77	

			**Pups**		

			PP20	PP30	ANOVA

**Mesenteric WAT**					
Weight g	C		0.14 ± 0.03	0.45 ± 0.04	T, TR
	OE		0.10 ± 0.02	0.31 ± 0.06 *	
Cell number n × 10^6^	C		189 ± 30.1	97.2 ± 6.51	TxTR
	OE		44.2 ± 15.1 *	169 ± 25.3	
Cell mass ng	C		0.72 ± 0.07	2.97 ± 0.55	TR, TxTR
	OE		4.65 ± 0.37 *	1.72 ± 0.23 *	
**Retroperitoneal WAT**					
Weight g	C		0.05 ± 0.002	0.24 ± 0.03	T
	OE		0.05 ± 0.001	0.16 ± 0.03 *	
Cell number n × 10^6^	C		16.4 ± 3.46	48.5 ± 10.3	T, TxTR
	OE		7.22 ± 1.24	18.1 ± 4.32 *	
Cell mass ng	C		3.41 ± 0.56	5.65 ± 0.71	T, TxTR
	OE		7.74 ± 1.14 *	8.03 ± 1.73	
**Perigonadal WAT**					
Weight g	C		0.05 ± 0.007	0.19 ± 0.02	T
	OE		0.03 ± 0.01	0.17 ± 0.04	
Cell number n × 10^6^	C		23.9 ± 4.80	41.7 ± 3.48	T, TR
	OE		11.7 ± 1.27	47.9 ± 5.64	
Cell mass ng	C		1.74 ± 0.29	4.41 ± 0.39	TR
	OE		2.55 ± 0.25	3.18 ± 0.25 *	
**Interscapular IBAT**					
Weight mg	C		138 ± 20.1	184 ± 11.7	TxTR
	OE		175 ± 17.4	147 ± 15.5	
Cell number n × 10^6^	C		74.1 ± 11.1	54.8 ± 7.47	
	OE		54.7 ± 6.65	45.5 ± 7.41	
Cell mass ng	C		1.77 ± 0.13	4.19 ± 0.55	T, TxTR
	OE		3.65 ± 0.31 *	2.62 ± 0.01	

Values are expressed as the mean ± s.e.m. of 6 animals. In pups the samples of perigonadal adipose tissue were a mixture of periovarian and epididymal tissue. Statistical analysis of the differences: Two-way ANOVA (time (T), treatment (TR) and interaction (TxTR)) was performed. Post-hoc Bonferroni test (comparison between C and OE): * = P < 0.01

## Discussion

The aim of this study was to determine the effects of OE treatment on pregnant rat metabolism; as expected, the treatment with OE decreased food intake and dampened increases in body weight during pregnancy. In fact, there is discordance between the magnitude of total food restriction and the weight loss this generates: i.e. a 10% food limitation seems too small to cause a nearly 50 % difference in weight increase (OE vs. controls) at the peak of pregnancy. This can be viewed as a confirmation that the slimming effects of OE are potent *per *se, i.e. not only because it decreases dietary energy availability; in fact, they are additive to those caused by food restriction, as has been shown using pair-fed [6] and in food-deprived lactation models [16]. Thus, the main effect of OE treatment was the observed decrease in different adipose tissue depots, since in late pregnancy the weight of individual adipose depots measured only 50% of those of controls. These data are validated by our balance energy analysis, in which the difference in total weight was a consequence of both a slower rate of protein accretion and a net loss of lipid. This maybe critical in the dams' catabolic context of the last third of pregnancy [17], when OE treatment selectively increased the catabolism of lipid and exerted only limited effects on protein metabolism and on the maintenance of glucose homeostasis, in a way similar to that previously described [2].

The mild effects of OE treatment on intermediate metabolism during pregnancy give support to the assumption of OE acting selectively on lipid stores. In addition, they establish a pattern different from that of the more aggressive food restriction models, in which normal development of the feto-placental unit depends on the extent of restriction [18]. This may help explain the important changes that pups of food restricted mothers undergo following delivery [19]. These changes, which may deeply determine their otherwise normal development [20] and body balance control in adulthood [21] are minor in our model. As a consequence, we have to assume that OE treatment causes a selective mobilization of maternal lipid stores in order to protect the near-normal development of the conceptus at the expense of mother's reserves. Since the differences in food intake of pregnant rats are maximal in late pregnancy, it is unlikely that the normal weight shown by pups at delivery will be a consequence of altered maternal food intake pattern; thus, the stable weight must be a consequence of OE treatment. Furthermore, this protective maternal pattern allows the normal growth of fetuses, resulting in smaller differences in weight at delivery, as well as in similar normal growth rates with controls up to weaning, a buffering model that differs completely from that described for the growth of pups from dams treated with OE during lactation [16].

Which mechanism induces this selective pattern? Although we cannot answer this question yet, the activation of apoptotic mechanisms by OE treatment in adipose tissue [22] may contribute to lipid mobilization. Moreover, OE treatment produces minor changes in the metabolic parameters of pregnant rats, as shown by the low levels of HDL-cholesterol and glucose, which were the only significant changes, observed in agreement with previous reports [2]. In addition, the maintenance of insulin levels under OE treatment reinforces the maintenance of insulin-glucose homeostasis in spite of decreased leptin and adiponectin levels. This fall in leptin during late pregnancy is consistent with previous results [23], and probably is a direct consequence of adipose tissue depletion. The decrease in leptin levels of lactating dams, however, may result from its suppressed release in response to food intake [24]. In addition, the decreased adipose depots suggest that OE may be ineffective in reducing this response.

It is possible that the differences in various adipose tissue sites reflect their metabolic specialization, since the metabolic activity of adipose tissue is not uniform nor is their cell mass and the presence of non-adipocyte cells [25]. Thus, while the retroperitoneal location has long been thought to serve as a storage site for readily usable lipids during the lactation period [26], our results show that during late pregnancy it remains undersized in the same proportion as the perigonadal or mesenteric sites; thus, contributing in a general way to the total loss of fat.

The consequences of OE treatment on pregnant rats were minimal, since after delivery they recovered their food intake levels up to weaning. However, OE-treated dams also exhibited the expected decrease in total and specific site lipid stores [16,23] resulting from the use of these lipids to sustain milk production.

The minimal differences in plasma parameters of pups from OE-treated rats vs. controls, and the minor changes observed in the weight gained up to day 20 after birth, are indicative of the maintenance of normal growth and unaffected metabolism. Thus, the low leptin levels observed in pups [23] must be a consequence of the initially limited adipose tissue masses that progressively grow thereafter.

Food intake restriction has been found to improve metabolic and functional parameters in rats [27]. Consequently, if this restriction is regarded as an ambiental condition suffered by pregnant rats, we can assume that the normal development of their pups would be affected. Thus, maternal OE treatment could induce selective signals that limit the normal growth of adipose tissue depots in their pups, thereby improving their chances of survival, and limiting the appearance of complications associated with the onset of obesity. These effects are specifically related to OE, since they are not paralleled by estrogen signaling: OE neither binds the estrogen receptor [5] nor increases body weight [28]. Furthermore, as OE treatment preserves body protein, any possible reduction in life span due to protein restriction *in utero *[29] is very probably minimized. These findings enhance the possible utilization of OE as experimental drug to prevent the development of obesity in later life.

## Conclusion

The pregnant rats can buffer the effects of OE treatment, mobilizing their own energy stores that can be used by their offspring, and generating simultaneously a metabolic imprinting in their pups that limits the accretion of adipose tissue, thereby reducing their pups' risk for developing obesity in adulthood.

## Competing interests

Dr. García-Peláez and Dr. Remesar are shareholders of a University-based spin-off: Oleoyl-Estrone Developments SL, created to develop structural analogous of the OE for their eventual application in the treatment of obesity. Oleoyl-Estrone Developments SL financed in part the study presented here in spite of not having potential interest as patentable material.

## Authors' contributions

BGP took part in animal handling and biochemical determinations, RV contributed to design the experience, analyze the data and to biochemical analysis, XR contributed to experimental design, animal managing and drafted the manuscript. All authors made contributions to the final version of manuscript. All authors read and approved the final manuscript.
